# Successful Transplantation of Multiple Organs from Donor after Helium Asphyxiation: First Case Report in Japan

**DOI:** 10.31662/jmaj.2024-0395

**Published:** 2025-03-21

**Authors:** Shunta Jinno, Takashi Hongo, Takafumi Obara, Tsuyoshi Nojima, Kohei Tsukahara, Tetsuya Yumoto, Hiromichi Naito, Atsunori Nakao

**Affiliations:** 1 Department of Emergency, Critical Care, and Disaster Medicine, Faculty of Medicine, Dentistry, and Pharmaceutical Sciences, Okayama University, Okayama, Japan

**Keywords:** brain death, heart arrest, helium

## Abstract

Helium inhalation has increased, but most cases are either minor injuries or deaths; there have not yet been any reported cases of brain death leading to organ donation. We report a patient who attempted helium inhalation and was declared brain dead and became an organ donor without complications. To the best of our knowledge, this is the first reported case of deceased organ donation following helium asphyxiation in Japan. The patient in cardiac arrest was found with a helium-filled vinyl bag sealed around the neck. During emergency medical transport to the hospital, a spontaneous return of circulation was obtained after 31 minutes of cardiopulmonary resuscitation. Upon hospital arrival, the physical examination revealed dilated pupils with no response to light. Electrocardiography showed widespread ST-segment depression and ST-segment elevation in augmented Vector Right, as well as elevated cardiac enzymes and decreased myocardial contractility. Head computed tomography revealed diffuse cerebral edema and loss of the gray-white matter boundary without signs of air embolism in the cerebral and coronary arteries. Despite comprehensive post-cardiac arrest care with recovery of organ function, brain death was confirmed on day 4 after hospitalization. The family consented to organ donation on the 11th day of hospitalization. The heart, lungs, liver, and two kidneys were successfully transplanted and all organs functioned. All organ grafts were functioning well at the 3-month follow-up. Our case demonstrates that brain death caused by helium inhalation is not a contraindication to organ donation.

## Introduction

Unfortunately, an increasing number of asphyxiations caused by helium-filled plastic bags have been widely reported in numerous countries ^(1)^. With increased accessibility of information and products via the Internet, we may encounter more patients affected by helium ^(2)^. Experimental research has convincingly shown that helium reduces ischemia-reperfusion damage in organ tissue ^(3)^. However, the organ-protective properties of helium might not yet be relevant for clinical practice. Although numerous case reports document individuals who have died from helium inhalation, no cases of brain death following helium inhalation resulting in organ donation have been reported ^(4)^. To the best of our knowledge, this represents the first documented case. Herein, we present a patient with brain death caused by anoxia from helium inhalation via a plastic bag placed over the head; resulting in the patient’s heart, lungs, liver, and kidneys being donated. In compliance with the ethical guidelines stipulated by the Revised Organ Transplant Act, all donor information has been fully anonymized. Specific details, including the donor’s gender, age, and the exact timing and location of the donation, have been deliberately omitted to ensure confidentiality. Ethical approval for this case report was obtained from the Japan Organ Transplant Network (JOTIRB-D-24006).

## Case Report

**Figure 1.**
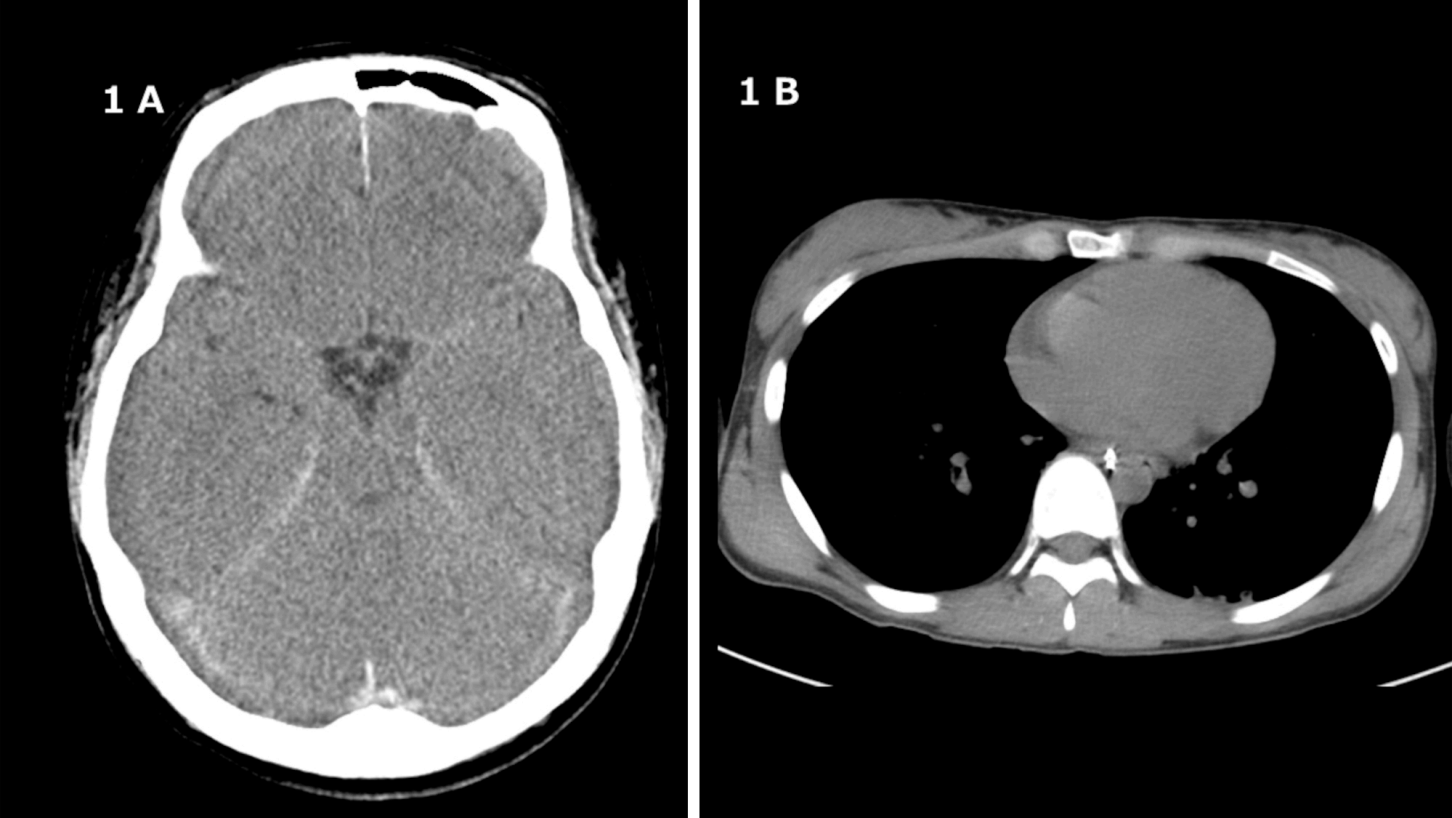
Head computed tomography shows diffuse brain edema and loss of gray-white matter contrast (A). There were no signs of air embolism in the cerebral (A) and coronary arteries (B).

The patient with no known past medical history was brought to our emergency department in cardiac arrest. The patient was found wearing a vinyl bag attached to a 100% helium gas cylinder sealed with duct tape at the neck, which contained no oxygen. A return of spontaneous circulation was obtained during transport after 31 minutes of cardiopulmonary resuscitation. An intratracheal intubation was performed by the emergency medical services personnel. Upon hospital arrival, the patient’s vital signs were as follows: Glasgow Coma Scale score of 3, heart rate of 99 beats per minute, blood pressure of 82/53 mmHg, Peripheral capillary oxygen saturation of 100% (with oxygen at 10 L), and dilated pupils with no response to light. Physical examination of the body was unremarkable. Routine toxicologic urine screening for drugs of abuse showed no anomalies. Electrocardiography showed widespread ST-segment depression and ST-segment elevation in aVR, as well as elevated cardiac enzymes and decreased myocardial contractility. Other laboratory tests were unremarkable as shown in Table 1. Head computed tomography revealed diffuse cerebral edema and loss of the gray-white matter boundary (Figure 1A). There were no signs of air embolism in the cerebral and coronary arteries (Figure 1B). Comprehensive management of post-cardiac arrest syndrome resulted in the recovery of cardiac function, and other laboratory parameters, including liver and renal function, remained within normal ranges throughout the intensive care unit stay. Despite intensive treatment, the patient was diagnosed with brain death based on serial neurological examinations, including electroencephalography on day 4. Through discussions with the patient’s family about end-of-life care, including organ donation, the relatives gave their approval for organ donation on the 11th day of hospitalization.

**Table 1.** Laboratory Data on Admission.

Arterial blood gas (10 L/min of oxygen)		Biochemistry	
pH	6.603	Total protein	5.3 g/dL
PaCO_2_	113.5 mmHg	Albumin	3.3 g/dL
PaO_2_	341.4 mmHg	Total bilirubin	0.32 mg/dL
HCO_3_^-^	10.5 mmol/L	AST	243 IU/L
Base excess	-30.1 mmol/L	ALT	132 IU/L
Lactate	18 mmol/L	Creatine kinase	107 IU/L
		BUN	9.6 mg/dL
**Complete blood count**		Creatinine	0.86 mg/dL
White blood cells	6640 /μL	Sodium	136 mEq/L
Hemoglobin	11.9 g/dL	Potassium	4.5 mEq/L
Hematocrit	40.1%	Chloride	100 mEq/L
Platelet count	25.1 ×10^4^ /μl	Glucose	248 mg/dL
		CRP	0.02 mg/dL
**Coagulation**		CK-Mb	53 IU/L
PT-INR	1.12		
APTT	79.8		
Fibrinogen	151 mg/dL		
D-dimer	9.9 μL/mL		

PT-INR, prothrombin time-international normalized ratio; APTT, activated partial thromboplastin time; AST, aspartate aminotransferase; ALT, alanine aminotransferase; BUN, blood urea nitrogen; CRP, C-reactive protein; CK-Mb, creatine kinase-myocardial band

**Figure 2.**
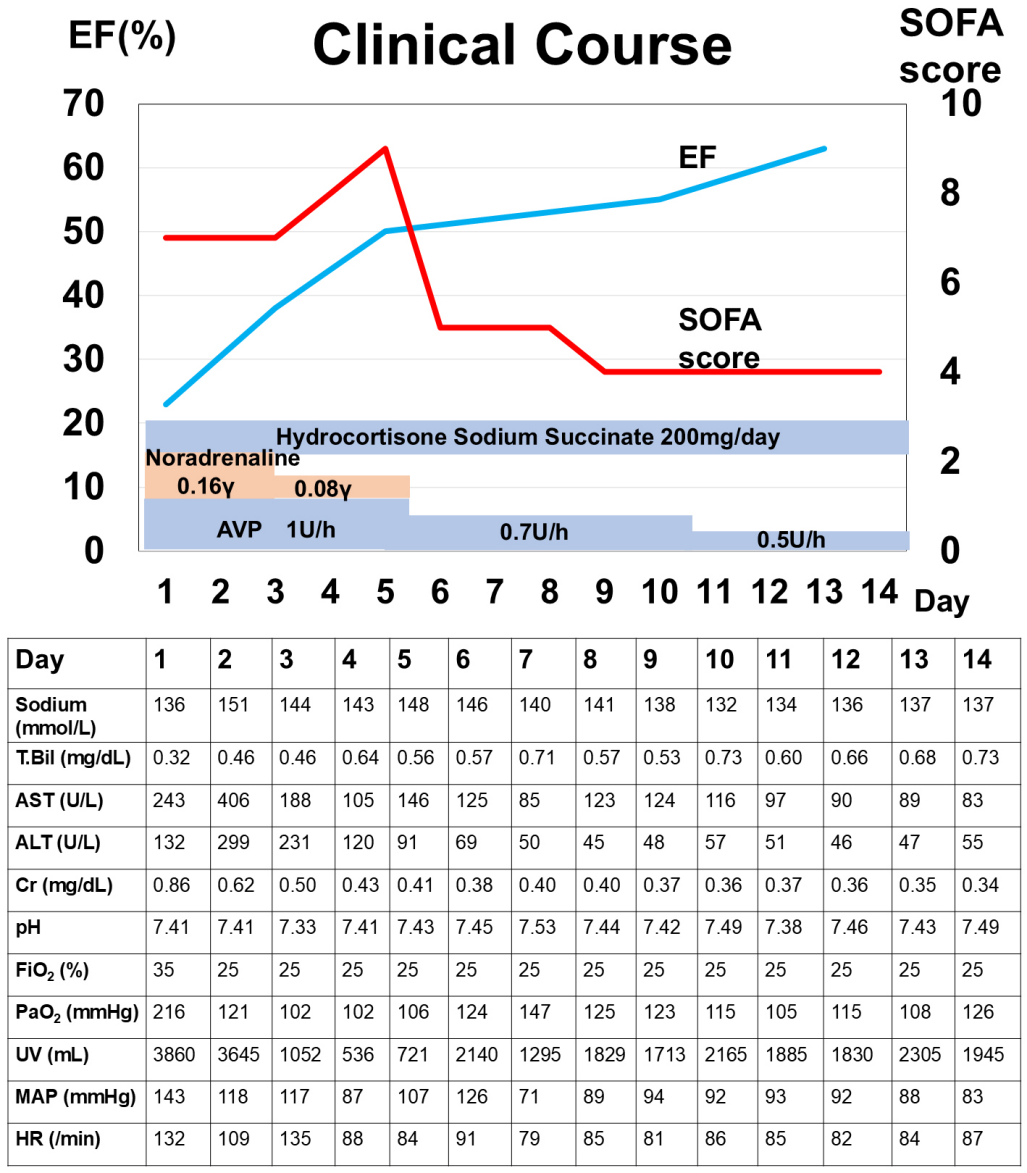
Clinical course of the patient. ^*^MAP and HR are documented daily at 7 a.m. as well as immediately after ICU admission and just before ICU discharge. AST: aspartate aminotransferase, ALT: alanine aminotransferase, AVP: arginine vasopressin, Cr: creatinine, EF: ejection fraction, HR: heart rate, ICU: intensive care unit; MAP: mean arterial pressure, SOFA: sequential organ failure assessment, T.Bil: total bilirubin, UV: urine volume

Figure 2 shows the clinical parameters of the patients. After the recovery of cardiac function, the heart, lungs, liver, and kidneys remained functional without evidence of organ failure throughout the course. The heart, lungs, liver, and kidneys were retrieved and transplanted into four recipients on the 14th day. Three months later, their clinical features and laboratory parameters improved considerably without complications.

## Discussion

In a 2022 retrospective cohort analysis, patients with asphyxia by gas inhalation were categorized into two groups based on the type of asphyxiating agent: simple and systemic ^(4)^. Helium is classified as a simple asphyxiant. Although helium is not directly toxic, its physical properties pose challenges. When inhaled, helium rapidly displaces atmospheric gases, notably carbon dioxide and oxygen.

In most mammals, the respiratory drive is triggered by excess carbon dioxide. Since helium also displaces carbon dioxide, the respiratory drive is weakened. As a result, inhaling pure helium leads to rapid unconsciousness, often resulting in death within minutes.

Brain dead patients who have attempted or committed suicide are generally considered suitable candidates for organ donation. Helium asphyxiation has been promoted as a non-violent, painless, and quick method of suicide ^(1)^. Challenges surrounding the diagnosis and management of brain death following suicidal asphyxiation with helium are similar to those in cases of suicide with other causes of brain death. Early recognition of brain death can prevent futile medical interventions, expedite organ transplantation, and allow closure for patients’ families.

The effects of helium on organ damage are not yet completely elucidated. Autopsy reports of suicide victims involving helium gas have indicated no evidence of organ-specific damage, with findings consistent with asphyxia ^(1)^. These reports suggest that helium intoxication does not directly cause organ-specific damage but instead induces hypoxemia. In our case, exposure to 100% helium likely resulted in brain death due to profound oxygen deprivation leading to asphyxiation. Of note, experimental research has demonstrated that helium surprisingly reduces ischemia-reperfusion damage in both the heart and brain ^(3)^. The exact mechanisms of helium-induced organ protection are not fully understood, but several signaling pathways have been identified ^(5)^. It is currently unknown whether the organ-protective effect of helium affects the function of transplanted organs. Accumulating cases and further investigation will be necessary to determine this in the future.

### Conclusion

Although solid organ transplantations from donors with helium inhalation have not been reported in the literature, helium asphyxiation is not a contraindication to organ donation.

## Article Information

### Conflicts of Interest

None

### Acknowledgement

We thank Christine Burr for editing the manuscript.

### Author Contributions

All authors meet the ICMJE authorship criteria.

Takashi Hongo, Shunta Jinno, Tetsuya Yumoto, Hiromichi Naito, and Atsunori Nakao: acquisition, analysis, and interpretation of data; Takashi Hongo, Shunta Jinno, Takafumi Obara, Tsuyoshi Nojima, Kohei Tsukahara, Tetsuya Yumoto, Hiromichi Naito, and Atsunori Nakao: in charge of the patient in our hospital; Takashi Hongo, Shunta Jinno, Tetsuya Yumoto, Hiromichi Naito, and Atsunori Nakao: conception and design of the study, analysis, and interpretation of data, and drafting or revision of the manuscript. The other authors have made substantial revisions and edits.

All authors have read and approved the final manuscript.

### Informed Consent

Written informed consent was obtained from the patient for the publication of this case report including the images.

### Approval of the Research Protocol

Not applicable.

### Registry and Registration of the Study/Trial

Not applicable.
